# Preparing Our Future Physicians: "We Will Reap What We Sow"

**DOI:** 10.7759/cureus.60799

**Published:** 2024-05-21

**Authors:** Larry Bush, Edlira Maska

**Affiliations:** 1 Department of Medicine, Wellington Regional Medical Center, Wellington, USA; 2 Department of Medicine, Charles E. Schmidt College of Medicine, Florida Atlantic University, Boca Raton, USA

**Keywords:** medical training, future physicians, clinical educators, residents, graduate medical education

## Abstract

As Designated Institutional Officer (LMB) and Program Director (EM) in a community teaching hospital, we are intimately involved with all aspects of Graduate Medical Education (GME) and find the rewarding part to consist of contributing towards the teaching of our future physicians, as well as the challenges imposed by the continuously evolving training requirements as set forth by the Accreditation Council for Graduate Medical Education (ACGME). While we are very aware of the standard training requisites that are put in place without exception for all accredited GME residency programs, whether they are part of a major (University) or minor (Community) teaching medical center, in this manuscript we are hoping to perhaps initiate a dialogue among clinical educators as to the future of graduate medical training, and how we as a medical community can commit to providing the best education experience for our residents, while preparing them to be the physicians our patients expect and deserve.

## Editorial

The world’s population is exponentially growing in leaps and bounds. What previously was once a compartmentalized and somewhat homogeneous group of like-individuals has evolved into a heterogeneously globalized society that comprises persons who in part are distinguished not only by their cultural backgrounds but increasingly more by their socioeconomic circumstances, much of which are almost predictable by the crest on the cover of their passports or the zip codes of their birthplace. To place this into numerical perspective, consider these facts. During the one hundred years spanning between 1923 and 2023, the world’s number of inhabitants has doubled three-fold, reaching today’s estimated global population of nearly eight billion persons [1]. In 1970, there were only one-half of today's population, which accounts for a staggering 7.8% of the total sum of persons who were ever born on this planet [2]. Although today’s United States (U.S.) population of approximately 336 million (many others not accounted for) encompasses only 4.3% of the world’s total, we are the world’s third most populous country gaining one person every 18 seconds (number of births + immigration - deaths) [3]. Furthermore, those >65 years old account for nearly one-fifth of the current U.S. census, which is twice the percentage of older persons in 1965. Concurrent with this rapidly increasing escalation in the number and age of the population is a mounting amount of medical knowledge as well as an explosive emergence of a sophisticated and expensive armamentarium of diagnostic and treatment modalities. All of this has led to the acute need for a significant expansion in the numbers of well-educated physicians, better access to qualified hospitals to accommodate medical school student clinical rotations, and perhaps most importantly, a growing number of adequate post-graduate residency training programs (GME). Self-declaration of quality alone is not an ample enough reason for top-level medical school graduates to want to apply to and select a particular GME program. Nor will it suffice if we honestly intend to achieve the level of practice and delivery of excellent, cost-efficient medical care that those we treat not only have come to expect but also rightfully deserve. Frankly spoken, we must be what we claim we are.

Without question, the recent increase in the numbers of advanced registered nurse practitioners (ARNPs) and physician assistants (PAs) has helped to alleviate the shortages of doctors by freeing up time previously spent on tasks equally carried out by non-physicians. However, such valued and appreciated allied health personnel are not, and should not be thought of as substitutes for a physician. Regardless of how the concept of physician oversight supervision is applied, any inclination to suggest otherwise is no more appropriate or acceptable than permitting an attorney’s paralegal to defend you in a complex legal court preceding or a hematology laboratory technologist to determine the specifics of a leukemic peripheral blood smear. Bluntly stated, “you don’t recognize what you don’t know”.

In the 10-year period between 2013 and 2023, the number of U.S. allopathic (MD) medical schools grew from 136 to today’s current number of 155 (there are an additional 41 osteopathic schools in 66 teaching locations in 35 states), effectively increasing the student enrollment in MD granting schools from 77,436 to 91,807, an 18.6% increase [4]. During the same period, there has been a 77% increase in the number of enrollees in osteopathic schools of medicine, now numbering greater than 35,000 students. It is very unlikely that there will be any considerable increase in the number of traditional university hospital residency training programs anytime soon in the foreseeable future. Therefore, to whom must the onus and responsibility of postgraduate medical and surgical resident training be relegated to? In 2017 there were a total of 10,090 Accreditation Council for Graduate Medical Education (ACGME) sanctioned programs (all specialties and subspecialties) in the U.S., charged with the task of preparing 130,545 residents and fellows for their careers in medicine. This has steadily increased during the past six years, such that currently there are 12,930 ACGME programs with 154,231 trainees, approximately one-quarter of which are international medical graduates (IMGs) [5]. In other words, one in seven physicians in the U.S. are presently in or entering training programs. As a result, most new residency training programs now function in community hospitals, many of which have come on board within the past decade. Several of these community hospital programs enjoy name association with one of the full-fledged universities. However, in general, the actual “academic” mingling between these institutions is sparse, with the university for all intent and purposes only loosely connected to the GME community program displaying its name on their letterhead. Nevertheless, often along with this name recognition comes the public perception of a greater degree of credibility and a higher level of standard of care, as well as the recognition as an “academic teaching center”. Yet, what exactly defines such centers warrants serious discussion.

Traditionally, it has been understood that medical institutions attained the title of an academic teaching center or institute by having qualified, experienced, and dedicated medical educators who in addition to administrative tasks spent much of their time teaching, mentoring, and engaging in clinical research and ongoing scholarly activities. To satisfactorily fulfill these requirements, it is imperative for the sponsoring institution to make certain that those selected to serve as both full-time and affiliated core and specialty faculty have been educated to do so, as well as granted adequate resources, are fairly compensated for their work, and afforded the necessary allotment of time to do the job well. As the designated institutional official (Larry M. Bush) and internal medicine residency program director (Edlira Maska) in a community hospital GME program, we are keenly aware of the fact that we are held to the same ACGME program accreditation standards as the university GME training programs. After all, there is one unifying goal for all ACGME-accredited programs, that of preparing future, competent physicians who can practice autonomously in any clinical setting.

It is the collective responsibility of the GME medical community, along with our medical centers and hospital partners to ensure that we diligently strive to turn these preconceived perceptions and expectations of the entering resident physicians, as well as the lay public surrounding such teaching hospitals, into reality. We view these as our vital moral and ethical obligations that must be fulfilled, as they are crucial to ensuring that those who follow behind us are provided the opportunity to complete their GME training with an educational experience, assurance, and preparation second to none. Allowing anything less cannot and should not be acceptable, as it risks the erosion of that which we consider to be the most noble and selective profession: the practice of medicine.

In ancient biblical terms, the phrase “we will reap what we sow” can be interpreted to mean; we will live with the results and consequences of our actions. We have confidence that the actions placed into motion will be the ones that years from now we will look back upon with pride and a deep sense of gratification. To borrow a quote, “indeed, if not now, when? if not by us, then by whom? as the future is not a gift, it is an achievement.”
